# A Method for Developing Novel 3D Cornea-on-a-Chip Using Primary Murine Corneal Epithelial and Endothelial Cells

**DOI:** 10.3389/fphar.2020.00453

**Published:** 2020-04-28

**Authors:** Jing Bai, Haojie Fu, Lauren Bazinet, Amy E. Birsner, Robert J. D'Amato

**Affiliations:** ^1^The Vascular Biology Program and Department of Surgery, Boston Children's Hospital, Harvard Medical School, Boston, MA, United States; ^2^Department of Mechanical Engineering, Massachusetts Institute of Technology, Cambridge, MA, United States; ^3^Department of Ophthalmology, Harvard Medical School, Boston, MA, United States

**Keywords:** microfluidic, primary cells, organ-on-a-chip, 3D cell-based models, cornea

## Abstract

Microfluidic-based organ-on-a-chip assays with simultaneous coculture of multi-cell types have been widely utilized for basic research and drug development. Here we describe a novel method for a primary cell-based corneal microphysiological system which aims to recapitulate the basic functions of the *in vivo* cornea and to study topically applied ocular drug permeation. In this study, the protocols for isolating and cultivating primary corneal epithelial cells and endothelial cells from mouse inbred strain C57BL/6J were optimized, to allow for the development of a primary-cell based microfluidic 3D micro-engineered cornea. This tissue unit, by overcoming the limitations of 2D conventional cell culture, supports new investigations on cornea function and facilitates drug delivery testing.

## Introduction

The cornea is an avascular and transparent mucosal tissue, and serves as one of the body's major mechanical barriers (DelMonte and Kim, 2011; Sridhar, 2018). Topical application of ocular drug to the cornea of the eye is the ideal route to treat diseases such as uveitis as well as retinal diseases, with maximal convenience and minimal invasiveness. It has been shown that topically administered antibody can quickly reach therapeutic levels in the anterior and posterior segment without the need for a penetration enhancer (Ottiger et al., 2009). As well, new approaches on ocular drug delivery, such as targeted drug delivery system (TDDS) (Xu et al., 2015) have accelerated the topical applications of liposome-, nanoparticle-, emulsion- and microspheres-based drug delivery, allowing for drugs to be concentrated on the target site with high efficacy and reduced side-effects (Kaluzhny et al., 2018). This may result in a shift of the current ocular drug delivery paradigm. However, developing an accurate corneal tissue models remains one of the major challenges to the study of drug permeation and delivery (Wright et al., 2020).

The human cornea is composed of 5 complex layers, namely, epithelium, the Bowman's layer, stroma, the Descemet's membrane and endothelium. The apical layer of epithelium contains tight junctions and is considered as one of the rate-limiting steps of topical administrations. Next, the stroma, composed of collagen fibers, serves as a diffusion barrier to lipophilic compounds. Lastly, a monolayer of endothelial cells also contributes to the restrictions on drug diffusion (Sosnova-Netukova et al., 2007). Certain drug-metabolizing enzymes and transporters which alter ophthalmological drug availability are present in the cornea (Shirasaki, 2008). Specifically, cornea primary cells express those important enzymes and transporters (Kaluzhny et al., 2018; Sridhar, 2018). Since these physiological features can be depleted during continuous *in vitro* passages, corneal primary cell-based, three-dimensional (3D) corneal models are critical to recapitulate unique physiological functions of the cornea.

Microfluidic-based 3D cell assays can mimic tissue/organ functions and cellular interactions, with the advantage of controllable geometrical, physical and biochemical microenvironment, and real-time imaging (Aref et al., 2013; Bai et al., 2015). This technology is emerging for *in vitro* testing platforms of ocular biological events (Guan et al., 2016; Seo et al., 2016; Bennet et al., 2018; Lu et al., 2019). There are previous studies reporting cornea-on-a-chip assays (Bennet et al., 2018; Seo et al., 2019) for testing drug delivery. However, it is still necessary to address the problem of drug diffusion within a controllable genetic background. Herein, we described a novel method to isolate and culture mouse primary corneal epithelial and endothelial cells, which are used to create a 3D microfluidic based cellular model.

In this study, mouse cornea was first dissected and epithelial/endothelial cells were isolated. Afterwards, the cells were cultivated separately in the two peripheral channels of a 3-channel microfluidic device with collagen matrix in the central channel to form a 3D model. To model the *in vivo* system, a condensed collagen layer was formed in the epithelium channel to mimic Bowman's layer with the concept of viscous finger (Bischel et al., 2012), a method to produce hydrogel lumen structure (Chin et al., 2002). This design is highly accessible to most of the standard biological labs and would provide a precise model to study physiological/pathological conditions of cornea tissues for ophthalmological drug discovery, potentially leading to development of novel ocular drug delivery methods across the anterior chamber.

## Materials and Equipment

### Reagents

#### Device Fabrication

Polydimethylsiloxane, PDMS, Dow Corning Sylgard 184 Silicone Elastomer base and curing agent (Ellesworth, Cat. No. 184).

#### Cell Culture

PCT Corneal Epithelium Medium, Low BPE (Zenbio, Cat. No. CnT-50).
Ham's F12 (Thermo Fisher Scientific, Cat. No. 11765047).
M199 (Thermo Fisher Scientific, Cat. No. 11150067).
DMEM GlutaMAX (Thermo Fisher Scientific, Cat. No. 10566-016).
HyClone Fetal Bovine Serum (Fisher Scientific, Cat. No. SH3007103).
1x insulin, transferrin, selenium (ITS) (Millipore-Sigma, Cat. No. I3146).
Ascorbic acid (Millipore-Sigma, Cat. No. A4403).
bFGF (STEMCELL Technologies, Cat. No. 78003.1).
1x Phosphate Buffer Saline (PBS), sterile.
1× anti-biotic/anti-mycotic solution in PBS.
10x PBS with Phenol Red.
1M NaOH in 1x PBS, sterile.
100% ethanol.
5% Bovine Serum Albumin.
Corning Matrigel Matrix (Corning, Cat. No. 354234).
Cell culture grade water.
Ethanol, 70% (vol/vol).
Dispase (Worthington, Cat. No. 9001-92-7).
Collagenase A (Sigma-Aldrich/Roche, Cat. No. 10103586001).
ACCUTASE™ (STEMCELL Technologies, Cat. No. 07920).
Corning^®^ Collagen I, Rat Tail (Corning, Cat. No. 354236).
Human Collagen Type IV (Sigma-Aldrich, Cat. No. C5533-5MG).

For immunofluorescent experiment (optional):

- Collagen-Fluorescein (FITC) Conjugate (Biovision, Cat. No. M1304-5).
- 4% Paraformaldehyde (PFA) (Sigma-Aldrich).
- 0.1% Triton-X (Sigma-Aldrich).
- ZO-1 polyclonal antibody (Invitrogen, Cat. No. 617300).
- Fluorescent dextran 70kDa Texas Red (Life Tech, Cat. No. D-1830).
- Fluorescent dextran 40kDa Texas Red (Thermo Fisher Scientific, Cat. No. D1829).
- Fluorescent dextran 10kDa (Thermo Fisher Scientific, Cat. No. D1828).
- K12 polyclonal antibody (Biorbyt, Cat. No. orb418611).
- Alexa Fluo 405 Goat anti-Rabbit IgG (H+L) (Invitrogen, Cat. No. A-31556).
- Alexa Fluo 594 Goat anti-Rabbit IgG (H+L) (Invitrogen, Cat. No. A-11037).
- Cell Tracker™ Red (Thermo Fisher Scientific, Cat. No.C34552).
- Cell Tracker™ Blue (Thermo Fisher Scientific, Cat. No.C12881).

### Equipment

Hemocytometer for cell counting.
Ophthalmic scissors, forceps.
CO2 Chamber for mouse euthanasia.
Stereomicroscope.
24-well tissue culture plate.
10-cm tissue culture dish.
0.45um syringe filter.
15ml Falcon Tube.
Scotch tape.
Glass coverslip.
Drying oven (60–80°C).
Vacuum desiccator.
Benchtop centrifuge.
Tissue culture incubator with 37°C and 5% CO2.
Water bath with 37°C.
Autoclave equipment.
Humid chamber prepared by autoclaved water filled in 1000µl pipet tip box for collagen gelation, kept in 37°C.
Plasma cleaner (Harrick Plasma, cat. no. PDC-001).
Confocal microscope.

## Methods

### Microfluidic Device Preparation

**Figure 1:**
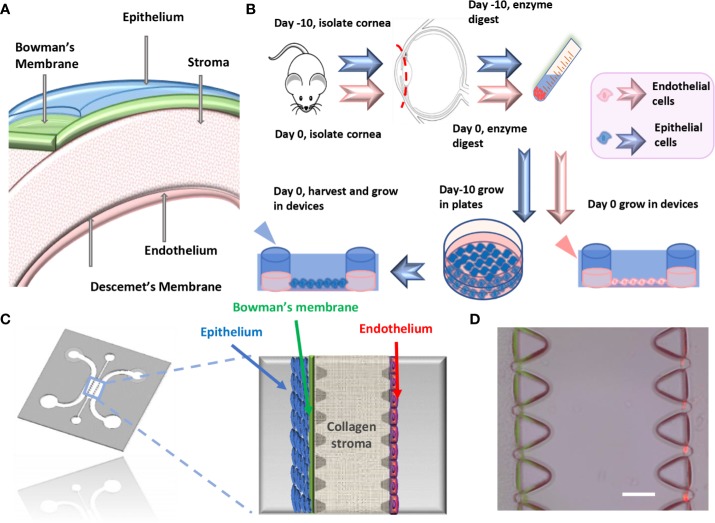
Microfluidic platform for cornea-on-a-chip. **(A)**. The structure of the cornea. **(B)**. Flow chart of mouse primary corneal endothelial cells/epithelial cell isolation and method for growing into devices, epithelial cells were cultured *in vitro* before being seeded into device while endothelial cells were isolated and cultured. **(C)**. Microfluidic cornea-on-a-chip device layout, red: endothelial cells, blue: epithelial cells. Corneal epithelium has 5-7 layers of cells and endothelium has a monolayer of cells, with Bowman's membrane represented as a condensed thin layer of green collagen gel. **(D)**. Representative phase contrast image for cornea-on-a-chip. Scale: 100μm.

Wafers were designed with AutoCAD and made by established SU-8 micropatterning methods (Shin et al., 2012; Levario et al., 2013) or by outsourcing. The wafer pattern contains 3 channels with one middle gel channel and two peripheral cell channels (**Figure 1**). To prepare the PDMS device, a disposable plastic cup was filled with Sylgard 184 silicone elastomer base and the curing agent in a 10:1 weight ratio. The solution was mixed and poured into a petri dish containing the SU-8 wafer and degassed for 40mins, before being transferred to a 70°C oven for 2h for curing. Afterwards, the PDMS negative pattern was carefully removed from the wafer and holes were punched through at the inlet- and outlet- of channels using dermal biopsy punches (1.5mm and 2.5mm). Scotch tape was used to remove small debris on the surface of device before autoclaving (120°C for 20 min). The sterile devices were dried in an oven at 80°C for at least 4 h and the devices were ready to bond on glass coverslip. The devices can be stored for up to 1 month at room temperature prior to bonding (Time: 8h).

On the subsequent day of preparation, glass coverslips were cleaned by immersing into 100% ethanol, air-dried, and then plasma treated together with the PDMS devices (pattern facing up). The treated surface was bonded together by applying light manual pressure. The bonded devices were kept in sterile dishes at 70°C oven overnight. The devices can be stored for up to 2 weeks at room temperature before seeding cells (Time: overnight).

### Isolation of Mouse Primary Corneal Epithelial and Endothelial Cells

#### Solution Preparing

- 0.8U/ml dispase and 1mg/ml collagenase A in DMEM was prepared separately and then filtered separately into individual 15ml tubes using syringes and 0.45um syringe filters, respectively.
- DMEM+10%FBS+1x pen/strep.
- 1× anti-biotic/anti-mycotic solution in 1x sterile PBS.
- 50 μg/ml collagen IV in 1x PBS.
- 10 ng/ml bFGF.
- 1:50 Matrigel in DMEM.
- Cornea endothelial cell culture media: F99 medium containing 1:1 Ham's F12 and M199, supplemented with 5% FBS, 20 μg/ml ascorbic acid, 1× ITS, 1× anti-biotic/anti-mycotic and 10 ng/ml bFGF (Li et al., 2007; Peh et al., 2013).
- PCT Corneal Epithelium Medium, Low BPE, prepared according to manufacturer's instruction.

#### Common Steps

All mouse experiments were conducted in compliance with the protocols approved by the Institutional Animal Care and Use Committee of Boston Children's Hospital (approval number 15-08-2998R for mouse experiments). Five C57BL/6J adult male mice (age 7-8 weeks) were euthanized by CO2, and intact eye globes were enucleated using a pair of fine curved forceps. The isolated eyes were placed into a 15ml tube containing 5ml of DMEM without serum and transferred to a new 10cm dish. Under a stereomicroscope, each eye was carefully dissected to eyecups by making a circumferential incision around the ora serrata (limbus), cut to include the limbus. Afterward, the lens was removed and posterior eye cup was discarded, leaving behind the cornea (time: 1.5 -2h).

The corneas were transferred to a new 15ml tube and washed three times in a 1× anti-biotic/anti-mycotic solution in PBS (wash buffer) for a total of 15 minutes. After wash buffer was removed, the corneas were resuspended in 6-7ml of serum free DMEM containing 0.8U/ml dispase. Corneas were then incubated for 1h at 37°C in water bath and the tube inverted every 15 min to ensure good digestion. Once the digestion has completed, enzyme solution was discarded by pipetting, and reaction was stopped by addition of 10ml of cold DMEM containing 10% FBS and 1x pen/strep. (time: 1h 30mins).

#### Corneal Epithelial Cells

Corneal epithelial cells were isolated by a combination of enzyme digestion and tissue explant methods. 5 wells of a 6-well plate were coated with 1:50 Matrigel for 1h at 37°C. Each digested cornea piece was carefully dissected equally into two pieces using ophthalmic scissors. The edge of curved cornea pieces was further cut halfway through to allow flat plating, and corneas from each mouse were attached in one well with epithelium facing up. Upon plating, one drop of media was applied on top of each corneal piece to prevent the tissue from drying out. 15 mins later, 2ml fresh corneal epithelium medium was added onto each well. Cells were grown without disturbance for 3 days before the corneal tissues were removed. Attached cells were grown to 80%-90% confluency for 5 to 7 days before accutase digestion. For digestion, 3ml accutase was applied to the cell culture for 10 min incubation. Cells were then harvested by washing with 6ml DMEM containing 10% FBS and 1x pen/strep. The suspension was then centrifuged at 800rpm for 5mins and cell pellet collected before growing into microfluidic devices (time: 8 -10 days).

#### Corneal Endothelial Cells

One day prior to seeding endothelial cells into microfluidic devices, the peripheral microfluidic channel was coated with collagen IV solution for at least 1h in 37°C incubator. Then the channels were washed twice with 100µl cell culture grade water and allowed to air-dry. The devices can be kept at room temperature for usage the following day (time: 1h).

On the subsequent day, 5 additional mice were euthanized and corneas were obtained and digested as previously described. Descemet's membrane and corneal endothelial cells were stripped from the posterior surface of the cornea under a stereomicroscope and suspended in 3-5ml of 1mg/ml collagenase A with a new 15ml tube and digested for additional 40mins in 37°C water bath. Afterward, the cell pellet was washed with 2-3ml 100% FBS and collected by centrifugation at 800rpm for 5 minutes before plating in the endothelial cell channel on the previously collagen IV-coated microfluidic device (time: 2h 30mins).

### Cornea-on-a-Chip Setup

#### Solution Preparation

- 200µl 2mg/ml and 4.5mg/ml collagen type-I, respectively, pH=7.2-7.4, was prepared separately by: a concentrated collagen type-I, sterile water, 10x PBS with phenol red to indicate the PH, 0.5M NaOH for pH adjustment (a pink-yellowish tone indicates a correct pH value). Keep all solutions on ice.
- 5% BSA dissolved in 1xPBS (for immunostaining, optional).
- Fluorescent dextran 70kDa, 40kDa and 10kDa Texas Red each 12.5μg/ml in DMEM (for diffusive experiment, optional).
- Cell Tracker™ Red and Blue, 1:200 diluted in serum free DMEM, separately (for visualization, optional).

#### Corneal Stroma and Bowman's Layer Formation

Up to 15 cornea-on-a-chip devices were able to be established with this protocol. For each device, the middle gel channel was filled with 10µl of 2mg/ml collagen type-I, pH=7.2-7.4 solution and the devices were put into a humid chamber in 37°C for 30 min to allow collagen gelation. To form the Bowman's layer, a condensed collagen gel thin layer was created along the epithelial side channel by viscous finger patterning. Briefly, 10µl of 4.5mg/ml collagen type-I solution was added in the epithelial cell channel and incubated in 37°C for 1min, before a droplet of DMEM with a volume of 50µl was added on from the 1.5mm diameter inlet. The less viscous fluid displaces the center of the collagen gel solution, leaving a thin lumen structure after the DMEM was removed. The device was incubated in 37°C incubator with humidity chamber again until polymerization was completed for 45mins. Then DMEM was removed and the devices were ready for cell seeding. (Time: 1h 30mins).

#### Cell Seeding in the Microfluidic Devices

Corneal epithelial and endothelial cells were harvested as described above. Endothelial cells were seeded with a density of 1x10^5^/ml (each device 30µl) and flipped 90° immediately to allow for the cells adhering to the stroma and an endothelial cell monolayer to be formed by gravity. The device was flipped back after 30mins and 70µl endothelial cell culture media was added to the media channel. Then the devices were left for another 30mins in 37°C incubator without disturbance. For epithelial cells, the seeding procedure was identical as with endothelial cells, except for the cell density at 3x 10^5^/ml and seeded twice. Epithelial cell culture media (CnT-50) was applied on this media channel. Cell culture media on both channels were changed daily for another 48h (Time: 48h).

#### Cell Immunofluorescent Staining

Primary corneal endothelial cells and epithelial cells were stained with ZO-1 (Invitrogen) and cytokeratin 12 (Biorbyt), respectively. The on-chip staining protocol could be found in our previous studies (Niu et al., 2014; Bai et al., 2015; Adriani et al., 2016). Briefly, cell culture media was removed from the side channels, and cells were washed with PBS once and fixed in 4% Paraformaldehyde (PFA) for 15min at room temperature. 0.1% Triton-X was used for membrane permeabilization for 15mins before blocking with 5% BSA dissolved in 1xPBS for 45mins-1h at room temperature. Afterward, cells were stained with primary antibodies (1:100) overnight at 4°C and secondary antibodies for 1h at room temperature. The secondary antibody (1:200) used in this study were Alexa Fluro 405 and Alexa Fluro 594 (goat anti-rabbit, Invitrogen). Fluorescent images were obtained by confocal microscopy (Olympus FV1000). For cell tracker staining experiments, epithelial cells and endothelial cells were incubated separately with blue and red solution for 15mins before rinsed by fresh DMEM. Then the cells were centrifuged and grown in the microfluidic devices.

#### Characterization of Corneal Model Permeability

60µl of fluorescent dextran at a concentration of 12.5 μg/mL (10kDa, 40kDa and 70kDa, Texas Red, respectively) was applied to the epithelial channel and allowed to flow across the stroma. Meanwhile, equal volume of non-fluorescent DMEM was applied through the endothelial cells channel to equate the pressure and to allow for dextran diffusion. Using fluorescence microscopy, the concentration fields in the endothelial channel were captured at 0min and 30mins, respectively. Their raw intensity profiles were analyzed using ImageJ (LOCI, University of Wisconsin). And diffusive permeability was calculated by

$$P_{D}=\beta \cdot D\frac{dC/dx}{\Delta C}$$

C = dextran fluorescence intensity, ΔC = the intensity drops across the vessel, β = area correction factor, dC/dx = slope of the concentration profile, D = dextran diffusion coefficient in collagen gel.

## Results and Discussion

In this study, we present a new method to develop a novel cornea-on-a-chip assay. Compared to established cornea-on-a-chip models, we have utilized isolated primary mouse corneal endothelial cells and epithelial cells in the setup. First, we have improved the current corneal epithelial cell culture protocol by an integration of tissue explant and enzyme digestion methods and accommodated these cells to grow in microfluidic chambers. Secondly, we have created the cornea-on-a-chip model with functional analysis *via* determining dextran diffusion permeability across the corneal barriers. The advantage of our model over the existing models is retaining *in vivo* characteristics, both genetically and phenotypically, to mimic corneal functions and drug diffusion processes. Thus, by utilizing rodent primary cells, it will better model genetic susceptibility to ocular diseases and enhance our understanding of genes function in a distinct genetic background. Furthermore, isolating rodent cells would allow for future on-chip studies on developmental and age-related ocular diseases.

### Isolation of Corneal Epithelial and Endothelial Cells

**Figure 2:**
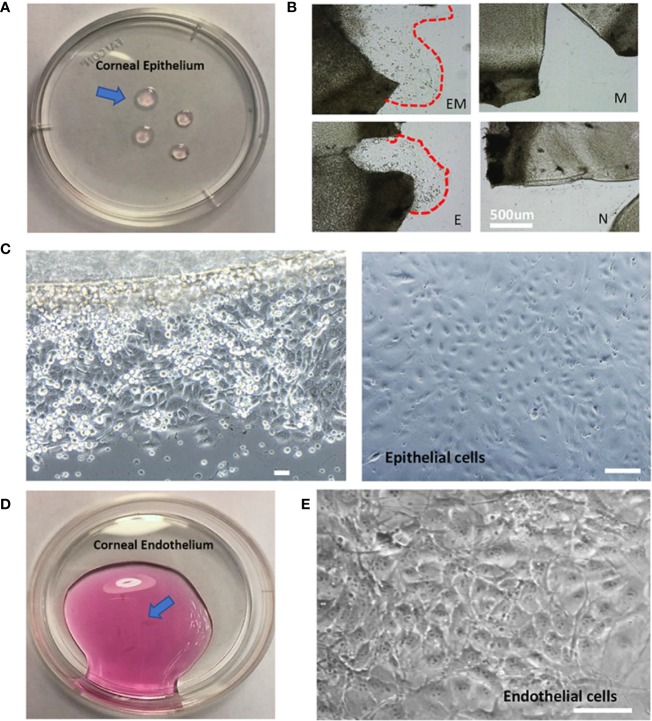
Morphology of isolated mouse corneal epithelial and endothelial cells. **(A)**. Isolated corneal epithelium (arrow). **(B)**. Comparison of four methods to culture corneal epithelial cells at day 3, EM = Enzyme digested+ Matrigel coated, E = Enzyme digested alone, M = Matrigel coated, N = No coating + no enzyme digestion. **(C)**. Epithelial cells cultured by tissue explant method at day 3 (left), day 10 (right). **(D)**. Isolated corneal endothelium (arrow). **(E)**. Morphology of corneal endothelial cells isolated and cultured. Scale: 200μm.

Cornea physiological structure was demonstrated in **Figure 1A** and a descriptive sketch on isolating corneal epithelial and endothelial cell procedure is shown in **Figure 1B**. To isolate corneal epithelial cells, the limbus where corneal epithelial stem/progenitor cells reside was isolated together with the corneal epithelial sheets (Li et al., 2017). These progenitor cells provide major resources and are critical for epithelial cell proliferation *in vitro*. The tissue explant method is a cell culture technique which involves growing cells *ex vivo* from pieces of tissue. Collagen or Matrigel coating is usually necessary for enhancing cell adhesion and migration. This technique has advantages in terms of cell numbers and viability over cell suspension culture (Ma and Liu, 2011), which tissue is completely digested into a single cell suspension by specific enzymes before being seeded into cell culture plates. In our method, we have further improved the current tissue explant method by an additional partial enzyme digestion step before the corneal tissue was attached and by growing this in a tissue culture plate (**Figure 2A**). Our results suggest that a combination of Matrigel plate coating with partial dispase digestion on the explanted cornea tissue (EM group) maximizes the yield of mouse corneal epithelial cells (**Figure 2B**). On day 4, significant increase in the cell numbers in the EM group was observed, conversely, no cells were migrating out in the groups without enzyme digestion. We have used the EM method throughout the study for culturing corneal epithelial cells.

After the corneal tissue was harvested, digested, and plated, epithelial cells began to migrate out onto the tissue culture plates within 3 days (**Figure 2C**, left). The cells display a cobblestone-like appearance and became confluent at day 10 (**Figure 2C**, right). The amount of epithelial cell harvested was up to 2.5x10^5^ cells in total. A previous study has optimized corneal epithelial cell culture by using CnT-50 media, a low-calcium, low bovine pituitary extract (BPE), serum-free medium (Kobayashi et al., 2009). Our findings also indicated that CnT-50 enables reproducible corneal epithelial cell expansion.

It is important to note that corneal endothelial cells are a type of slow-cycling cell and their proliferation *in vitro* is minimal. Therefore, in our study, we were only culturing the *in vitro* single endothelial cell suspension into microfluidic devices to form endothelium monolayer. 10 mouse corneas were able to yield 5x10^4^ live endothelial cells in total. Extracted corneal endothelium (**Figure 2D**) and phase-contrast images indicated that corneal endothelial cells retained their unique hexagonal cellular morphology (**Figure 2E**).

### Cornea-on-a-Chip Development

**Figure 3:**
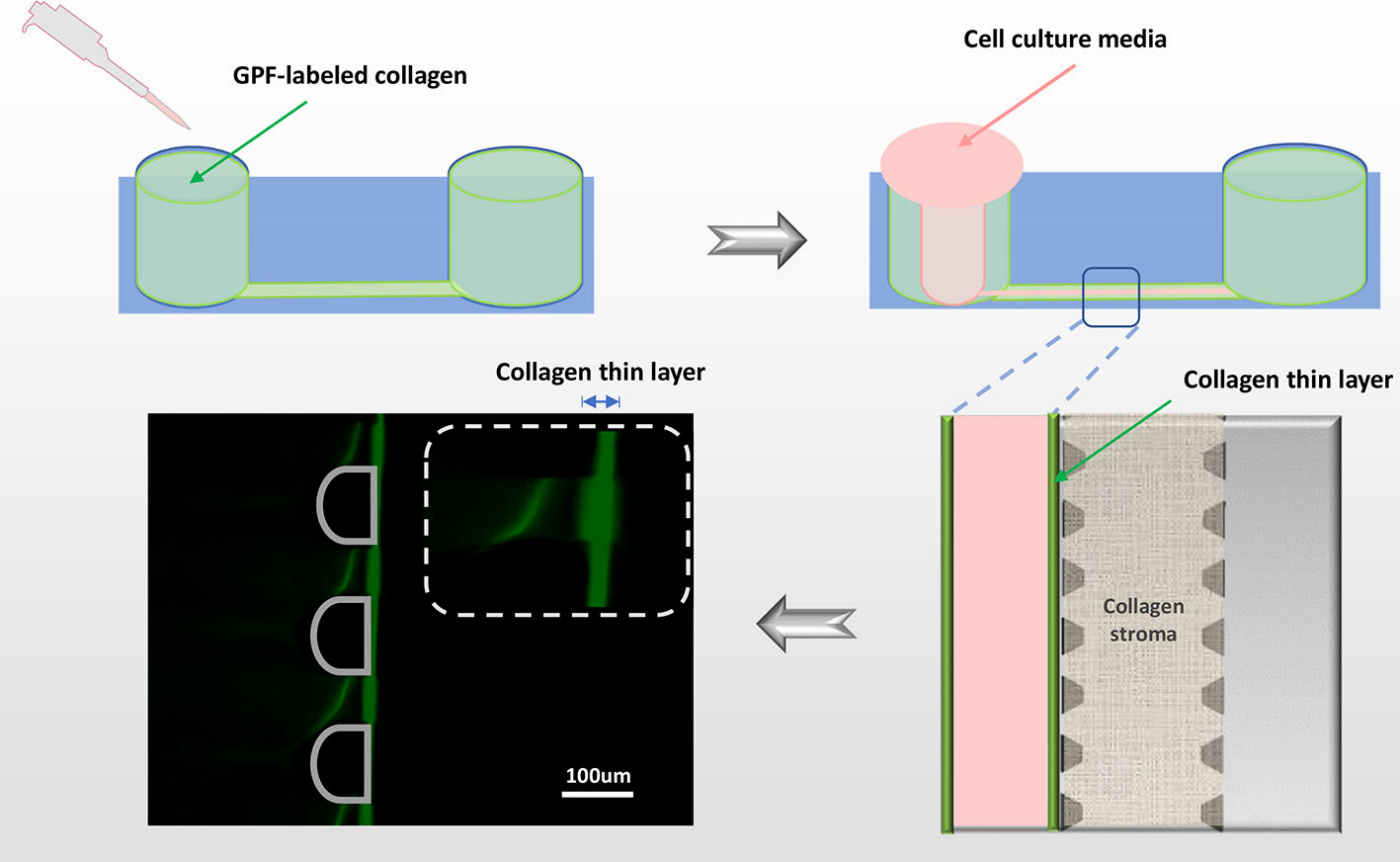
Viscous finger patterning to assemble Bowman's membrane. The side microchannel is filled with 4.5mg/ml collagen I solution, and incubated in 37°C for 1min before applying DMEM media to the same channel from one inlet. A lumen is formed by DMEM displacing the collagen solution in the center of the microchannel, leaving a thin layer of condensed collagen (green fluorescent line) which mimics the Bowman's membrane. To demonstrate the collagen thin layer, GFP-labeled collagen was mixed with non-fluorescent collagen in 1:10. Green: collagen; pink: cell culture media.

The microfluidic cornea-on-a-chip setup was shown in **Figure 1C**, and a representative phase-contrast image was shown in **Figure 1D**. It recapitulates the corneal tissue structures with an epithelial layer and an endothelial layer on the two peripheral channels. A 3D collagen gel matrix (2mg/ml) is formed in the middle channel representing corneal stroma which forms the bulk of the cornea framework and comprises 80%–85% of its thickness (DelMonte and Kim, 2011). Bowman's membrane has critical functions for drug transport and diffusion. It acts as a thick supporting collagen matrix layer for the epithelium with a thickness of 17.7 ± 1.6 μm (Sridhar, 2018). In this study, a denser but thinner collagen layer (4.5mg/ml), formed by viscous finger patterning, is designed to mimic the Bowman's membrane. Viscous finger patterning is designed with the concept of a less viscous fluid replacing a more viscous fluid and thereby forming “finger-like” projections of the less viscous fluid (Walker and Beebe, 2002; Bischel et al., 2012). **Figure 3** has illustrated the viscous finger patterning process. Microchannels were 160μm tall and 400μm wide. A droplet of culture media with a volume of 50μl would create enough surface energy and pressure to pump the liquid through a microchannel with 1.5mm inlet diameters and 2.5 mm outlet diameters.

**Figure 4:**
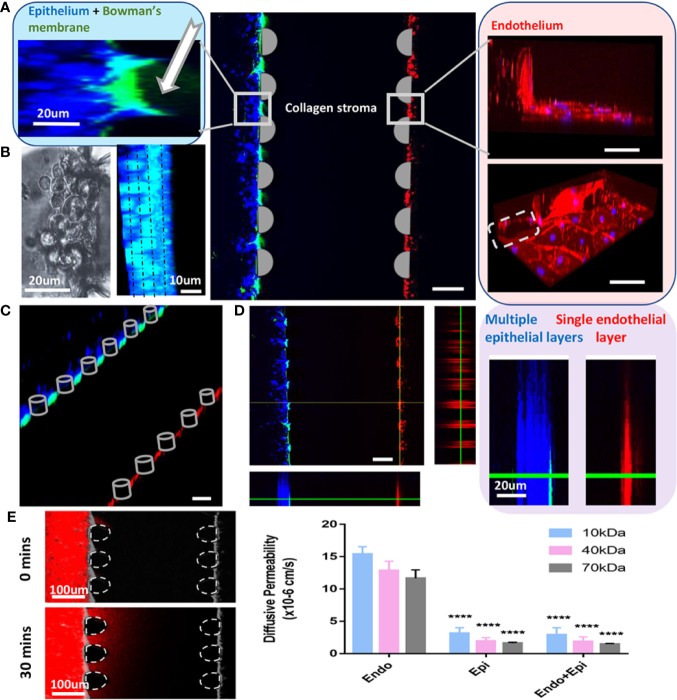
Cornea-on-a-chip setup. **(A)**. Immunofluorescent image of the device structure and cells 24h after seeding; middle: a 3D projection view of the cornea-on-a-chip assay; left: higher magnification on the corneal epithelial side, Bowman's layer as GPF-labeled condensed collagen (white arrow), epithelial cells (blue); right: endothelial cells monolayer was stained by ZO-1 (red), nuclei (blue), gray dotted line: PDMS pillar in the microfluidic device. **(B)**. Phase-contrast (left) and immunofluorescent image (right) on corneal epithelial cells stained with cytokeratin 12 at 48h after seeding. Cells form 4-5 layers as indicated by dotted line. Blue: cytokeratin 12, cyan: nuclei. **(C)**. Immunofluorescent surpass view image showing the overlay of the cornea-on-a-chip setup. **(D)**. A 3-section view (left) and a comparison of epithelial multiple layer and endothelial monolayer (right). Endothelial cells were labeled with CellTracker™ Red and epithelial cells were labeled with CellTracker™ Blue. Bowman's membrane was shown in GFP-labeled collagen. **(E)**. Dextran diffusion assay for 10kDa, 40kDa and 70kDa dextran label with Texas red and diffusive permeability was calculated from the fluorescent intensity across epithelial, endothelial layer and the entire cornea. Left, an example of dextran intensity images for the entire cornea at 0min and 30mins, respectively. Right, diffusive permeability in three conditions for three dextran molecules: epi = only epithelial cells present; endo = only endothelial cells present; endo+epi = co-culture of endothelial and epithelial cells. All data are expressed as mean ± S.E.M. Comparison between multiple groups was performed by one-way-ANOVA and indicated by following Tukey means comparison tests (defined as: ****p < 0.0001). Analysis performed by Prism 7 (GraphPad). Scale:100um.

After collagen gelation, DMEM was removed from the microchannel and corneal epithelial cells were seeded on top of the Bowman's membrane which formed cell layers in 48h. We observed that epithelial cells continued to grow in the microfluidic chips and formed a structure of 5-7 cell layers [**Figures 4A** (left), **B, D**(right)], which mimics the corneal epithelial sheets *in vivo*. Cytokeratin 12 immunostaining for corneal epithelial cells was shown in **Figure 4B** (left: phase contrast image; right: immunofluorescent image). For the endothelium, immunofluorescent staining on the intact monolayer is shown in **Figures 4A** (ZO-1, right) and **D** (right). A surpass view as well as a 3-section view of the setup was shown in **Figures 4C, D** (left). The established microfluidic platform was functional through two weeks after seeding.

To further validate the model, we have performed dextran diffusion assay with 3 different molecular weights (10kDa, 40kDa and 70kDa) to determine the diffusive permeability in this model. The purpose of this assay was to mimic drug diffusion across the corneal barrier (**Figure 4E**). With or without endothelial cell monolayer, there was a minimal difference on permeability for all three dextrans. However, in the absence of epithelial cell clusters, a significant increase in the permeability indicated that epithelium is the major determinant of drug diffusion transportation rates. The results are in agreement with previous findings (Bennet et al., 2018).

In this study, we have described a 3D microfluidic-based cell assay to recapitulate the basic corneal functional unit. The prototype allows for the study of corneal function/pathology as well as ocular drug diffusion into the anterior chamber.

## Limitation of This Study

The microfluidic setup has advantages over 2D cell culture assays in terms of a small scale and important 3D features. However, this study has limitations to be considered. Normal corneal epithelium is a 5–6 layers structure with three types of cells: superficial cells, wing cells, and basal cells (Sridhar, 2018). It is difficult to distinguish and isolate individual epithelial cell subtypes and to restructure them *in vitro*. In addition, while the predominant component of the stroma is collagen type I, type VI and type XII. This study did not address the differences of the various collagen types.

## Data Availability Statement

The datasets generated for this study are available on request to the corresponding author.

## Ethics Statement

The animal study was reviewed and approved by Institutional Animal Care and Use Committee of Boston Children's Hospital.

## Author Contributions

JB, RD'A designed research. JB, LB, and HF performed research and analyzed data. JB wrote the manuscript. JB, RD'A, HF, and AB contributed to the preparation of the manuscript.

## Funding

This study was supported, in part, by the NIH National Eye Institute under Award Number R01EY012726-12 (to RD'A).

## Conflict of Interest

The authors declare that the research was conducted in the absence of any commercial or financial relationships that could be construed as a potential conflict of interest.
